# *Aiouea altomacaensis* (Lauraceae), new species from the mountains of Rio de Janeiro, Brazil

**DOI:** 10.3897/BDJ.14.e193820

**Published:** 2026-05-22

**Authors:** Marcelo Leandro Brotto, Pedro Luís Rodrigues de Moraes

**Affiliations:** 1 Museu Botânico Municipal de Curitiba, Curitiba, Brazil Museu Botânico Municipal de Curitiba Curitiba Brazil; 2 Universidade Estadual Paulista (UNESP), Rio Claro, Brazil Universidade Estadual Paulista (UNESP) Rio Claro Brazil

**Keywords:** biodiversity, *
Cinnamomum
*, Neotropics, *
Ocotea
*, plant taxonomy

## Abstract

**Background:**

The Brazilian Atlantic Rainforest is one of the centres of diversity for the genus *Aiouea* (Lauraceae). This Neotropical genus comprises approximately 85 species, 35 of which occur in Brazil, with 24 being endemic. Based on a taxonomic review of herbarium collections and recently gathered specimens, we propose a new species of *Aiouea* endemic to the country and the Atlantic Rainforest.

**New information:**

Herein, we describe and illustrate *Aiouea altomacaensis*. It is a medium-sized tree endemic to the State of Rio de Janeiro, southeast Brazil, specifically restricted to the mountainous region between Nova Friburgo and Petrópolis. This taxon can be confused with three others: *Aiouea acarodomatifera*, *A. albopunctata* and *A. ombrophila*. *Aiouea altomacaensis* differs from these mainly by its narrow leaves and short inflorescences. Initially, the species was assessed as Endangered (EN) under IUCN Criteria due to its restricted distribution. However, Data Deficient (DD) is a more appropriate classification until more reliable information on its actual range becomes available.

## Introduction

*Aiouea* Aubl. is a Neotropical genus of Lauraceae comprising species with 2- and 4-locular anthers, following the circumscription by Rohde et al. (2017). Under this proposal, 85 species are recognised with distribution from northern Argentina through Central America and the West Indies to Mexico. Of these, 35 species occur in Brazil, with 24 being endemic (Li et al. 2025, van der Werff 2025). In the country, the greatest species richness is concentrated in the Cerrado and Atlantic Rainforest domains.

The most recent taxonomic treatments for the genus in the Atlantic Rainforest were provided by Renner (1982) for 2-locular anthers species and Lorea-Hernández (1996) for 4-locular anthers, with the latter still circumscribed within *Cinnamomum*. At that time, incomplete or doubtful specimens were left undetermined. Consequently, we conducted targeted field efforts to relocate these taxa. We were successful collecting the first flowering specimen of one of these dubious trees. This material provides the morphological evidence necessary to propose a new species of *Aiouea*. The description of this taxon, alongside other recent discoveries, such as *Aiouea albopunctata* Brotto, *Aiouea ombrophila* Brotto & Völtz and *Aiouea myrmecophila* Brotto & P.L.R.Moraes (Brotto 2022, Brotto and Völtz 2024, Brotto and Moraes 2025), strengthens the status of the Atlantic Rainforest as a centre of diversity for the genus.

This underscores the significant research potential of the Atlantic Rainforest, which continues to yield new taxa, such as the one that we have discovered and are proposing in the present study. We provide a formal description including line drawings, information about distribution and habitat and an IUCN extinction risk assessment.

## Materials and methods

This study was based on morphological analyses of herbarium material, including new samples collected by us, as well as a review of literature on related species. The scientific collections consulted were the following: ALCB, B, BHCB, BM, BR, C, CAP, CEPEC, CESJ, CGE, CVRD, EFC, ESA, ESAL, F, FLOR, FUEL, FURB, G, G-DC, GH, GOET, GZU, HBG, HCF, HBR, HRCB, HUEFS, HUFU, IAC, IAN, ICN, INPA, IPA, K, KIEL, L, LE, MBM, MBML, MEL, MG, MO, NY, OXF, P, R, RB, S, SP, SPF, SPSF, U, UEC, UPCB, US, VIC, VIES and W (acronyms according to Thiers (2026) [continuously updated]). Collections viewed exclusively through digital images: C, GH, HUFU, L, S, U, US and W.

The assessment of the species extinction risk has been based on IUCN Red List Categories and Criteria (IUCN 2024). The Extent of Occurrence (EOO) and Area of Occupancy (AOO) were estimated using GeoCAT software (Bachman et al. 2011). The AOO was calculated based on 4 km² cells. Four points were used to calculate EOO and AOO. Three vouchers lacked geographic coordinates; thus, their coordinates were georeferenced, based on locality descriptions. The coordinates we inferred are as follows: *Góes 116* (22°31'48''S, 43°12'38''W), *Lima 3547* (22°25'55''S, 42°30'56''W), *Pessoa 469* (22°22'39''S, 42°29'10''W).

Some descriptive characteristics follow these criteria: terminal buds, branchlets and young leaves refer to structures formed in the last few months, which differ in colour, indumentum and consistency from mature ones. Adult structures, however, remain unchanged for years. Scent was classified into three quality categories (pleasant, unpleasant and indistinct) and three intensity levels (strong, moderate and weak).

The illustrations were produced as follows: The line drawings were prepared with ink pen on tracing paper, using a stereomicroscope. Photographs of live material were taken using a NIKON D5200 camera. The map was constructed using original and inferred geographic coordinates from all vouchers, as explained previously. All figures were finalised in Photopea® software. Abbreviations used in the text are as follows: fl. = flower; immat. fr. = immature fruit; fr. = fruit; st. = sterile.

## Taxon treatments

### Aiouea altomacaensis

Brotto & P.L.R.Moraes sp. nov.

5C2DCDE5-2DBB-5898-A63D-D93BA8341A3B

77380584-1

#### Materials

- **Type status:** Holotype. **Occurrence:** catalogNumber: HRCB 82002; recordNumber: 6197; recordedBy: M.L. Brotto; reproductiveCondition: fl.; occurrenceID: A46CC33B-6838-5534-90A1-90195C6D7A30; **Location:** country: Brazil; stateProvince: Rio de Janeiro; municipality: Nova Friburgo; locality: Parque Estadual dos Três Picos, Macaé de Cima, trilha para o Pico do Faraó; minimumElevationInMeters: 1193; verbatimLatitude: 22°26′08.9″S; verbatimLongitude: 42°32′42.4″W; **Event:** year: 2025; month: 4; day: 9; **Record Level:** institutionCode: HRCB
- **Type status:** Isotype. **Occurrence:** catalogNumber: EFC025472; recordNumber: 6197; recordedBy: M.L. Brotto; reproductiveCondition: fl.; occurrenceID: B59F0637-ABBE-5F4E-8764-7C37E70BE8B8; **Location:** country: Brazil; stateProvince: Rio de Janeiro; municipality: Nova Friburgo; locality: Parque Estadual dos Três Picos, Macaé de Cima, trilha para o Pico do Faraó; minimumElevationInMeters: 1193; verbatimLatitude: 22°26′08.9″S; verbatimLongitude: 42°32′42.4″W; **Event:** year: 2025; month: 4; day: 9; **Record Level:** institutionCode: EFC
- **Type status:** Paratype. **Occurrence:** catalogNumber: BHCB 24542; recordNumber: 3547; recordedBy: *H.C. de Lima*; reproductiveCondition: immat. fr.; occurrenceID: C10A7A05-5584-50AE-B167-91759470C0FC; **Location:** country: Brazil; stateProvince: Rio de Janeiro; municipality: Nova Friburgo; locality: Reserva Ecológica Municipal de Macaé de Cima, Nascente do Rio das Flores; **Event:** year: 1989; month: 4; day: 20; **Record Level:** institutionCode: BHCB
- **Type status:** Paratype. **Occurrence:** catalogNumber: MBM 445816; recordNumber: 3547; recordedBy: *H.C. de Lima*; reproductiveCondition: immat. fr.; occurrenceID: 90C7B4EA-8FD2-588A-8E9C-1CD6DE259F38; **Location:** country: Brazil; stateProvince: Rio de Janeiro; municipality: Nova Friburgo; locality: Reserva Ecológica Municipal de Macaé de Cima, Nascente do Rio das Flores; **Event:** year: 1989; month: 4; day: 20; **Record Level:** institutionCode: MBM
- **Type status:** Paratype. **Occurrence:** catalogNumber: MO-264248; recordNumber: 3547; recordedBy: *H.C. de Lima*; reproductiveCondition: immat. fr.; occurrenceID: 29B4B9A3-8CFB-536A-8E56-00EC87EECDCF; **Location:** country: Brazil; stateProvince: Rio de Janeiro; municipality: Nova Friburgo; locality: Reserva Ecológica Municipal de Macaé de Cima, Nascente do Rio das Flores; **Event:** year: 1989; month: 4; day: 20; **Record Level:** institutionCode: MO; source: immat. fr.
- **Type status:** Paratype. **Occurrence:** catalogNumber: NY00459853; recordNumber: 3547; recordedBy: *H.C. de Lima*; reproductiveCondition: immat. fr.; occurrenceID: 6922A947-84E1-5501-957D-810F4A434963; **Location:** country: Brazil; stateProvince: Rio de Janeiro; municipality: Nova Friburgo; locality: Reserva Ecológica Municipal de Macaé de Cima, Nascente do Rio das Flores; **Event:** year: 1989; month: 4; day: 20; **Record Level:** institutionCode: NY
- **Type status:** Paratype. **Occurrence:** catalogNumber: RB00128668; recordNumber: 3547; recordedBy: *H.C. de Lima*; reproductiveCondition: immat. fr.; occurrenceID: 1995417F-35B6-5582-A2CE-2F0E3B662B9A; **Location:** country: Brazil; stateProvince: Rio de Janeiro; municipality: Nova Friburgo; locality: Reserva Ecológica Municipal de Macaé de Cima, Nascente do Rio das Flores; **Event:** year: 1989; month: 4; day: 20; **Record Level:** institutionCode: RB
- **Type status:** Paratype. **Occurrence:** catalogNumber: SP054427; recordNumber: 3547; recordedBy: *H.C. de Lima*; reproductiveCondition: immat. fr.; occurrenceID: 69658CCD-B43B-5D8B-AF0F-90DF19F85826; **Location:** country: Brazil; stateProvince: Rio de Janeiro; municipality: Nova Friburgo; locality: Reserva Ecológica Municipal de Macaé de Cima, Nascente do Rio das Flores; **Event:** year: 1989; month: 4; day: 20; **Record Level:** institutionCode: SP
- **Type status:** Paratype. **Occurrence:** catalogNumber: SPSF 16668; recordNumber: 3547; recordedBy: *H.C. de Lima*; reproductiveCondition: immat. fr.; occurrenceID: ED1A6F8F-C0B9-58AA-82F5-79ECCD190CDB; **Location:** country: Brazil; stateProvince: Rio de Janeiro; municipality: Nova Friburgo; locality: Reserva Ecológica Municipal de Macaé de Cima, Nascente do Rio das Flores; **Event:** year: 1989; month: 4; day: 20; **Record Level:** institutionCode: SPSF
- **Type status:** Paratype. **Occurrence:** catalogNumber: BHCB 24543; recordNumber: 469; recordedBy: S.V.A. Pessoa; reproductiveCondition: immat. fr.; occurrenceID: 489BA273-3786-5407-A0BD-70FAACA7EA5E; **Location:** country: Brazil; stateProvince: Rio de Janeiro; municipality: Nova Friburgo; locality: região de Macaé de Cima, estrada para o Sítio Sophronites, depois da porteira; **Event:** year: 1989; month: 8; day: 3; **Record Level:** institutionCode: BHCB
- **Type status:** Paratype. **Occurrence:** catalogNumber: MO-264257; recordNumber: 469; recordedBy: S.V.A. Pessoa; reproductiveCondition: immat. fr.; occurrenceID: 965B8C4E-1F70-56BE-B6D2-CB489662A361; **Location:** country: Brazil; stateProvince: Rio de Janeiro; municipality: Nova Friburgo; locality: região de Macaé de Cima, estrada para o Sítio Sophronites, depois da porteira; **Event:** year: 1989; month: 8; day: 3; **Record Level:** institutionCode: MO
- **Type status:** Paratype. **Occurrence:** catalogNumber: NY00459855; recordNumber: 469; recordedBy: S.V.A. Pessoa; reproductiveCondition: immat. fr.; occurrenceID: B327181D-CE3A-5048-81ED-5D401FB30001; **Location:** country: Brazil; stateProvince: Rio de Janeiro; municipality: Nova Friburgo; locality: região de Macaé de Cima, estrada para o Sítio Sophronites, depois da porteira; **Event:** year: 1989; month: 8; day: 3; **Record Level:** institutionCode: NY
- **Type status:** Paratype. **Occurrence:** catalogNumber: RB00128659; recordNumber: 469; recordedBy: S.V.A. Pessoa; reproductiveCondition: immat. fr.; occurrenceID: 4164056F-B450-54C8-8691-EA33D20ED729; **Location:** country: Brazil; stateProvince: Rio de Janeiro; municipality: Nova Friburgo; locality: região de Macaé de Cima, estrada para o Sítio Sophronites, depois da porteira; **Event:** year: 1989; month: 8; day: 3; **Record Level:** institutionCode: RB
- **Type status:** Paratype. **Occurrence:** catalogNumber: SP054426; recordNumber: 469; recordedBy: S.V.A. Pessoa; reproductiveCondition: immat. fr.; occurrenceID: AD0726E3-B4E4-59AE-BDBC-ED2834D00D40; **Location:** country: Brazil; stateProvince: Rio de Janeiro; municipality: Nova Friburgo; locality: região de Macaé de Cima, estrada para o Sítio Sophronites, depois da porteira; **Event:** year: 1989; month: 8; day: 3; **Record Level:** institutionCode: SP
- **Type status:** Paratype. **Occurrence:** catalogNumber: SPSF 16580; recordNumber: 469; recordedBy: S.V.A. Pessoa; reproductiveCondition: immat. fr.; occurrenceID: 9268ADED-D43F-5886-A739-4F2AB6D279D2; **Location:** country: Brazil; stateProvince: Rio de Janeiro; municipality: Nova Friburgo; locality: região de Macaé de Cima, estrada para o Sítio Sophronites, depois da porteira; **Event:** year: 1989; month: 8; day: 3; **Record Level:** institutionCode: SPSF
- **Type status:** Paratype. **Occurrence:** catalogNumber: MBM446160; recordNumber: 5601; recordedBy: M.L. Brotto; occurrenceID: C7C5D264-1EDE-5C4F-872B-3A221A820737; **Location:** country: Brazil; stateProvince: Rio de Janeiro; municipality: Nova Friburgo; locality: Parque Estadual dos Três Picos, Macaé de Cima, trilha para o Pico do Faraó; minimumElevationInMeters: 1193; verbatimLatitude: 22°26′09″S; verbatimLongitude: 42°32′42″W; **Event:** year: 2023; month: 10; day: 28; eventRemarks: st.; **Record Level:** institutionCode: MBM
- **Type status:** Paratype. **Occurrence:** catalogNumber: MBM331650; recordNumber: 116; recordedBy: O.C. Góes; reproductiveCondition: immat. fr.; occurrenceID: 9E1EBC52-6C8A-5172-A4C0-1121FE57BA92; **Location:** country: Brazil; stateProvince: Rio de Janeiro; municipality: Petrópolis; locality: Quitandinha; **Event:** year: 1948; **Record Level:** institutionCode: MBM
- **Type status:** Paratype. **Occurrence:** catalogNumber: RB00133418; recordNumber: 116; recordedBy: O.C. Góes; reproductiveCondition: immat. fr.; occurrenceID: 762A2DAD-E17A-5A0E-9CD8-46E8E68C643C; **Location:** country: Brazil; stateProvince: Rio de Janeiro; municipality: Petrópolis; locality: Quitandinha; **Event:** year: 1948; **Record Level:** institutionCode: RB

#### Description

**Figure 1.**
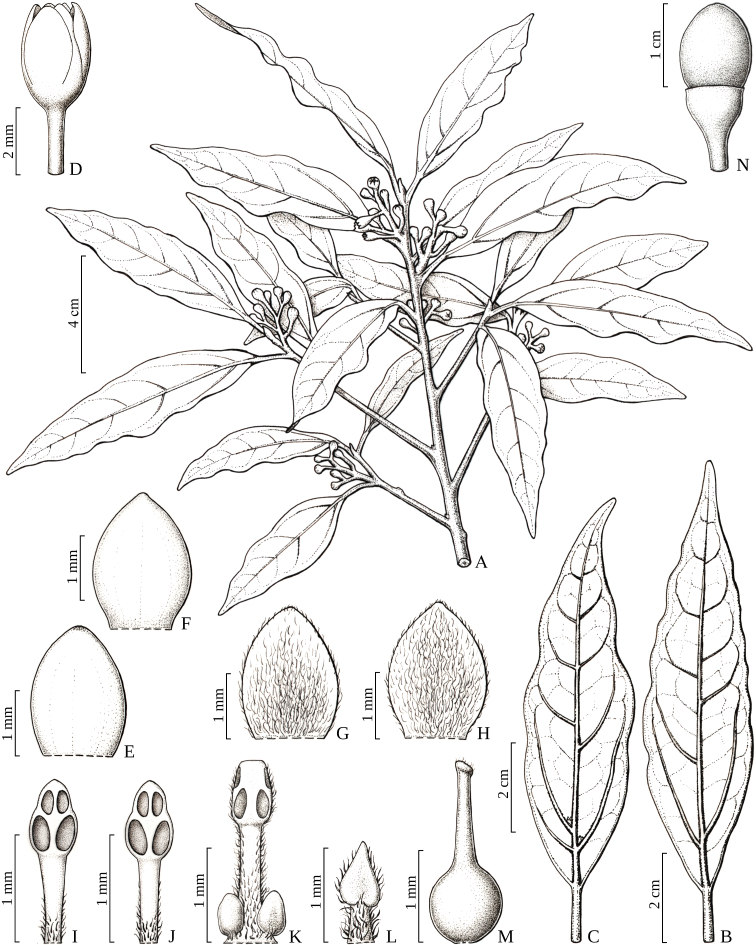
*Aiouea altomacaensis* Brotto & P.L.R.Moraes. **A** flowering branch; **B** adult leaf (abaxial surface); **C** young leaf with domatia (abaxial surface); **D** flower; **E** abaxial tepal surface whorl I; **F** abaxial tepal surface whorl II; **G** adaxial tepal surface whorl I; **H** adaxial tepal surface whorl II; **I** stamen surface whorl I, adaxial side; **J** stamen surface whorl II, adaxial side; **K** stamen surface whorl III, abaxial side; **L** staminode surface whorl IV, adaxial side; **M** pistil; **N** cupule and fruit (A–M. Brotto 6197; N. Pessoa 469).

**Figure 2.**
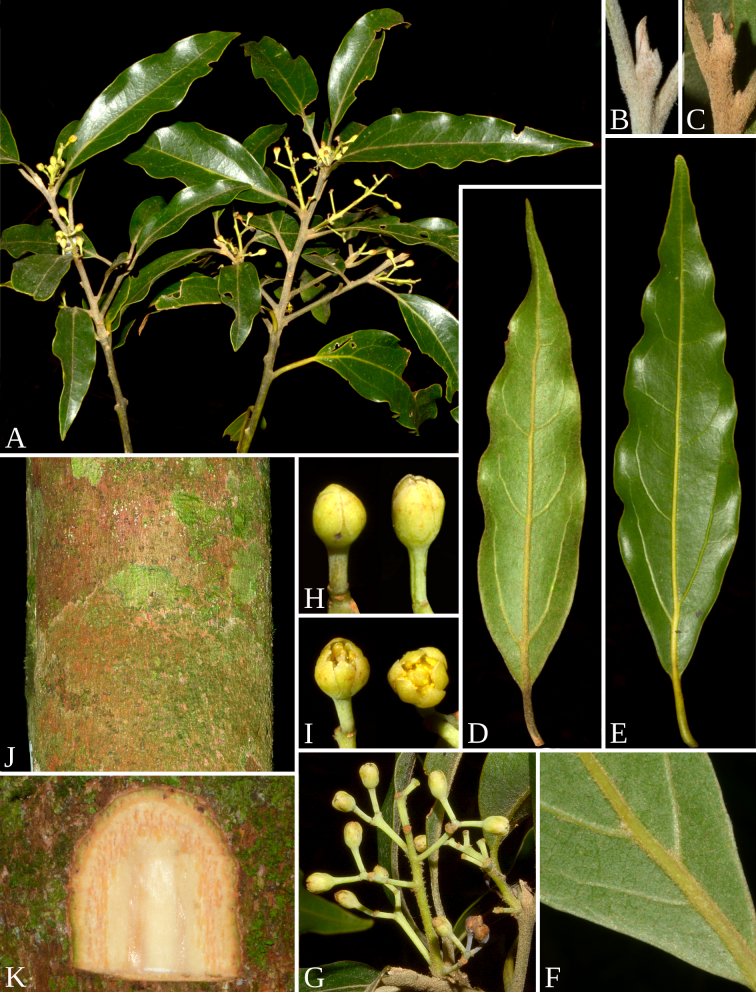
*Aiouea altomacaensis* Brotto & P.L.R.Moraes. **A** flowering branch; **B** terminal bud and young branchlet; **C** terminal bud and adult branchlet; **D** young leaf (abaxial surface); **E** adult leaf (abaxial surface); **F** base leaf with domatia (abaxial surface); **G** inflorescence; **H** floral bud and flower at anthesis; **I** flowers at anthesis; **J** rhytidome of a thick trunk; **K** detail of rhytidome. Photographs by M.L. Brotto (voucher A, C−K. Brotto 6197, B. Brotto 5601).

Trees up to 16 m tall. Trunk cylindrical; bark brown, homogeneous, both rough and lenticellate, lenticels round, small; slash bright-beige, tangential section heterogeneous with fibres light-brown, odorous, with a pleasant, moderately intense smell. Branches terete, brown-greenish, glabrous; young branchlets compressed, cinereo-tomentose; terminal buds cinereo-tomentose; adult branchlets and terminal buds cinnamomeo-tomentose. Leaves alternate, patent; petioles 0.6–1.8 cm long, slightly canaliculate, adult glabrous, young cinereo-tomentose; blades (3.8)6.0–10.0(11.0) × (0.9)1.3–2.3(3.8) cm, narrow lanceolate to lanceolate, chartaceous; margin undulate and thickened; base acute, apex acute or slightly acuminate; adaxial surface glabrous, young cinereo-puberulous, midrib immersed at the base and flat to the apex, secondary veins flat, reticulation moderately dense, inconspicuous, not ampullaceous at domatia; abaxial surface glabrous, young cinereo-puberulous, midrib raised, 0.6–1.0 mm thick, secondary veins 5–7 on each side, first pair raised, 0.3–0.4 mm thick, 20–30° to midrib, other secondary veins raised, 30–55° to midrib, reticulation conspicuous; venation pinnate to subtripliveined, brochidodromous to weakly eucamptodromous; domatia in the axils of the first pair of secondary veins or lacking, ± flat, covered with cinereous hairs. Inflorescences axillary, small paniculate-cymose, 1.4–2.6 cm long, with up to 12 flowers, glabrescent, rachis compressed. Flowers yellow-greenish *in vivo*, glabrous, 2.0–2.2 mm diam.; pedicel 2.0–3.1 mm long; receptacle 0.5–0.7 mm long, subhemispherical, inconspicuous, outside glabrous, inside tomentose; tepals 6, subequal, oval-elliptic, erect at anthesis, whorl I 2.0–2.4 × 1.2–1.4 mm, outer surface glabrous, inner surface puberulous, just less dense towards the margin, whorl II 2.2–2.4 × 1.4 mm, outer surface glabrous, inner surface tomentose, minutely papillose at the tip; stamens 9, anthers 4-locular, filaments longer than the anthers, whorl I 1.6–1.7 mm long, filament 0.8–1.0 mm long, puberulous at the basal half, anther 0.7 × 0.4 mm, ovate-elliptic, apex obtuse, locelli introrse, whorl II 1.5–1.7 mm long, filament 0.8–1.0 mm long, puberulous at the basal half, anther 0.5 × 0.4 mm, ovate-elliptic, apex obtuse, locelli introrse, whorl III 1.9–2.0 mm long, filament 1.4–1.5 mm long, tomentose, glands 0.4 mm diam. present at base, ovate, anther 0.5 × 0.4 mm, ovate-rectangular, apex truncate, upper locelli lateral, lower locelli lateral-extrorse, staminodia of whorl IV 1.2–1.4 mm long, filament tomentose, tip triangular-sagittate; pistil 2.0–2.1 mm long, glabrous, ovary globose ca. 0.8 mm, style 1.2 mm, stigma simple with hairs or none. Fruits ellipsoid, 1.1 × 0.8 cm (immature fruit: Pessoa 469); cupules obconic, 0.9 × 0.6 cm, covering 1/6 of the fruit, tepals deciduous (Figs 1, 2).

#### Diagnosis

*Aiouea altomacaensis* most closely resembles *A. albopunctata* in its vegetative morphology and tree architecture. *Aiouea altomacaensis* differs from the latter mainly by its smaller inflorescence of 1.4–2.6 cm length (vs. 3.0–13.0 cm), with up to 12 flowers (vs. up to 60), smaller flowers measuring 2.0–2.2 mm in diameter (vs. 2.7–3.0 mm), shallow receptacle, subhemispheric, inconspicuous (vs. deep, obconic, conspicuous), filaments of stamens of whorl III 2–3× longer than anthers (vs. 1–1.5×) and short obconic cupule of 0.9 × 0.6 cm (vs. 1.6 × 1.0 cm), that covers 1/6 of the fruit (vs. 1/3 to 1/4).

#### Etymology

Most specimens originate from Macaé de Cima, a locality situated at the headwaters of the Macaé River. This region was historically known as Alto Macahe until the 19^th^ century. Consequently, the specific epithet is derived from this ancient toponym, while adopting a modernised spelling.

#### Distribution

**Figure 3.**
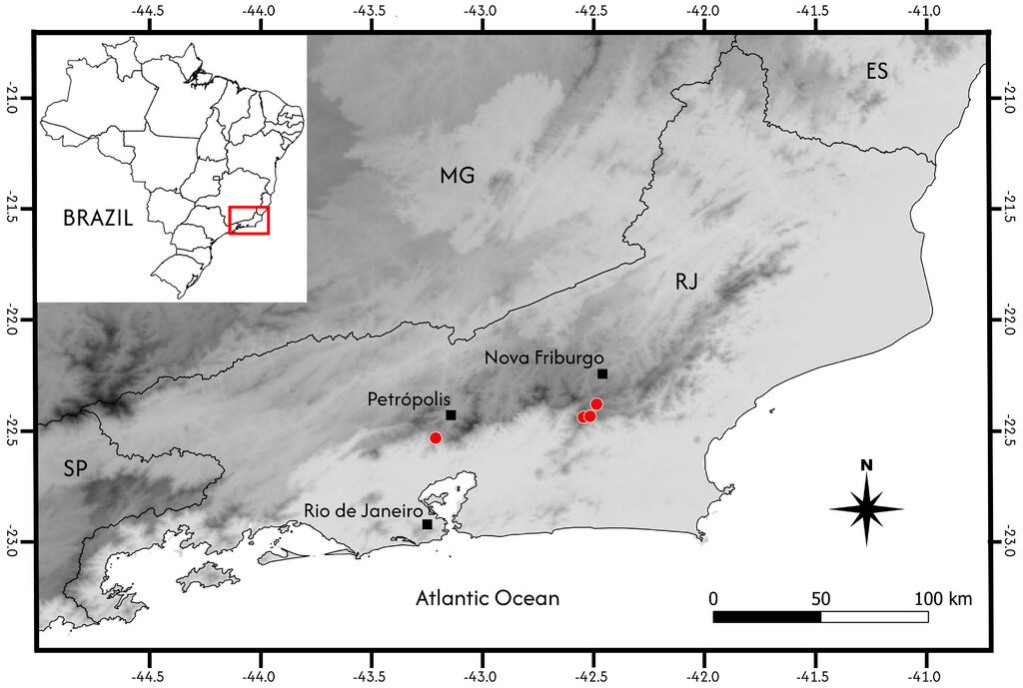
Distribution map of *Aiouea altomacaensis* Brotto & P.L.R.Moraes (red dots). Legend: Espírito Santo (ES), Minas Gerais (MG), Rio de Janeiro (RJ), and São Paulo (SP). Elevation scale: Light grey = slightly above sea level; dark grey = up to 2500 m elevation.

*Aiouea altomacaensis* has been recorded in only two municipalities in Rio de Janeiro State, south-eastern Brazil: Nova Friburgo and Petrópolis (Fig. 3).

#### Ecology

It occurs within Atlantic Rainforest fragments along the Serra do Mar mountain range at elevations between 900 and 1200 m. This region receives the State's highest rainfall, with annual averages ranging from 2500 to 2800 mm (Silva and Dereczynski 2014). According to the Brazilian vegetation classification system (IBGE 2012), the area is characterised as Floresta Ombrófila Densa (Ombrophilous Dense Forest). Its primary characteristics include a high density of trees, a continuous canopy and the absence of a dry season throughout the year.

#### Conservation

The samples are distributed across a restricted area within the mountainous region of Rio de Janeiro, with a maximum distance of only 76 km between the two most distal points. This, combined with the small number of samples, results in an EOO of 200 km² and an AOO of 16 km². The conclusion would be that the species is Endangered (EN). Despite that assessment, we consider the distribution of this species to be wider than currently documented. The Serra do Mar region maintains high habitat integrity, yet the upper slopes remain under-investigated. Therefore, we follow a precautionary approach by classifying it as Data Deficient (DD). It is important to note that the holotype represents the only specimen collected from a legally protected area, specifically from within Parque Estadual dos Três Picos.

#### Taxon discussion

Amongst the species within the genus occurring in the Atlantic Rainforest, three exhibit the highest morphological similarity and tree habit, as listed in Table 1. The new species most closely resembles *Aiouea albopunctata* Brotto in the appearance of its leaves and trees architecture. Both have leaves that are quite similar in size, shape, consistency, number and angle of secondary veins and reticulation. However, *Aiouea altomacaensis* has lanceolate leaves, mostly narrow, with raised secondary veins that gradually increase in angle relative to the midrib as they approach the apex. The petioles are slightly shorter, measuring 0.6–1.8 cm long. Furthermore, they have a patent (horizontal) arrangement in the tree canopy. Its branches and young leaves are densely covered with ash-grey hairs. The leaves turn dark green when they become mature and lose almost all of their haris. On the other hand, *Aiouea albopunctata* has leaves that tend to be slightly wider, elongate-elliptic, elliptic or lanceolate, with only the first pair of secondary veins raised, the others slightly raised. In addition, the second pair and the more distal ones form a much less acute angle with the midrib than the first pair of secondary veins. The petioles are slightly longer, 1.2–2.0 cm and the trees have canopy with pendulous leaves. It has ferruginous pubescent branches. The young leaves are reddish-orange, turning green when they mature.

The two species can be easily distinguished by their inflorescence, flower, cupule and fruit. *Aiouea albopunctata* has inflorescences that can reach up to 13 cm in length and contain up to 60 flowers. The flower has a larger diameter (2.7–3.0 mm), the receptacle is obconic and conspicuous and the filaments are slightly longer (1.2–1.5×) than the anthers of whorl III. It has a larger fruit (1.3 × 0.9 cm) with a robust hemispherical cupule (1.6 × 1.0 cm). In contrast, *Aiouea altomacaensis* has small inflorescences measuring a maximum of 2.6 cm and containing up to 12 flowers. Its flower has a smaller diameter (2.0–2.2 mm), the receptacle is subhemispheric and inconspicuous and the filaments are much longer (3×) than the anthers of whorl III. The species has a slightly smaller fruit (1.1 × 0.8 cm) and smaller obconic cupule (0.9 × 0.6 cm).

Furthermore, the two species are parapatric. *Aiouea albopunctata* inhabits inland forests with a seasonal climate, whereas the new species occurs closer to the Atlantic coast in an ever-wet climate without a dry season.

*Aiouea acarodomatifera* Kosterm. also bears a close resemblance to the new taxon. Some specimens may have leaves of the same size, shape and similar venation. However, in most specimens, the leaves are wider or smaller, with an elliptic or ovate shape, always with the adaxial surface ampullaceous over the domatia. In addition to this, the midrib is raised on abaxial surface and the secondary veins are slightly raised. The first pair of secondary veins has a smaller thickness of 0.1–0.3 mm. In contrast, *Aiouea altomacaensis* has consistently narrow leaves, with a narrow lanceolate or lanceolate shape, and a non-ampullaceous over the domatia. The midrib and secondary veins are raised and the first pair of secondary veins has a thickness of 0.3–0.4 mm.

Inflorescence and floral characters are more diagnostic, facilitating the distinction between the two species. *Aiouea acarodomatifera* has a relatively longer inflorescence (1.8–8.0 cm). Its flower has a larger diameter (3.0–4.0 mm), the receptacle is obconic and conspicuous, all anthers have 2 locules and the filaments are slightly longer (1–2×) than the anthers of whorl III. In living material, the flowers are greenish-white. In contrast, *Aiouea altomacaensis* has a smaller inflorescence (1.4–2.6 cm), smaller diameter of flower (2.0–2.2 mm), 4-locular anthers, longer filaments (3×) and yellowish-green flowers.

These species are sympatric. The type of *A. acarodomatifera*, voucher *Nunes 201* (barcodes MO-4686966; NY01104817; RB00539142; RB00570800; SP000814), is from Governador Portella-Monte Sinai, now Miguel Pereira, a municipality adjacent to Petrópolis.

*Aiouea ombrophila* Brotto & Völtz is another species that shares the same habitat and resembles the new taxon. In general, this species has broad leaves; narrow leaves (lanceolate) are less common, as in voucher *Brotto 4805* (barcode CESJ082358; barcode MBM440276; barcode SP004697; HRCB 79553; MBML 59185; SPSF 58717). Its petiole is longer (1.5–2.6 cm) and the blade is usually oval-elliptic, broadly elliptic or elliptic. In contrast, *A. altomacaensis* has a narrow lanceolate or lanceolate blade and a shorter petiole (0.6–1.8 cm).

The best characteristics to differentiate the two are inflorescence, flower, cupule and fruit. *Aiouea ombrophila* has a larger inflorescence (6.0–12.0 cm) with up to 45 flowers, a flower with larger diameter (3.0–3.5 mm), conspicuous obconic receptacle, filaments are slightly longer (1–1.7×) than the anthers of whorl III and bear large (0.9 mm diam.), elliptical-cubic glands in this whorl. In turn, *A. altomacaensis* has smaller inflorescence (1.4–2.6 cm), smaller diameter of flower (2.0–2.2 mm), longer filaments (3×) and small glands (0.4 mm), of ovate shape. Furthermore, the first species has a larger, turbine-shaped cupule (2.3 × 1.2 cm) and a larger fruit (2.0 × 1.2 cm), while the second has a short, obconic cupule (0.9 × 0.6 mm) and a smaller fruit (1.1 × 0.8 cm).

The narrow leaves and venation pattern of *A. altomacaensis* lead to a strong resemblance to *Ocotea catharinensis* Mez and *Ocotea porosa* (Nees & Mart.) Barroso, explaining why the voucher *Góes 116* (barcodes MBM331650; RB00133418) was originally determined as *O. porosa*. Both possess small staminodia of whorl IV compared to the new species, which has 1.2–1.4 mm long and triangular-sagittate tip. Of the two, only *O. catharinensis* is sympatric, being much more frequent in the area compared to *A. altomacaensis*. In addition, *O. porosa* and *O. catharinensis* have well-developed pit-domatia lacking in *A. altomacaensis*.

#### Phenology

Flowering specimen were collected in April; fruiting specimens were collected in April and August.

## Supplementary Material

XML Treatment for Aiouea altomacaensis

## Figures and Tables

**Table 1.** Comparative morphology of *Aiouea altomacaensis* Brotto & P.L.R.Moraes and related species.

**Characters**	***A. altomacaensis***	***A. acarodomatifera***	***A. albopunctata***	***A. ombrophila***
Habit	Tree (16 m)	Tree (18 m)	Tree (30 m)	Tree (30 m)
Rhytidome colour	brown	grey	greyish-brown	pale-yellow
Rhytidome texture	rough, uncracked	rough, uncracked	fissured-scaly, scales woody	scales woody
Petiole length (cm)	0.6–1.8	0.5–1.4	1.2–2.0	1.5–2.6
Leaf shape	narrow lanceolate or lanceolate	elliptic or ovate	elongate-elliptic, elliptic or lanceolate	oval-elliptic, broadly elliptic, elliptic or lanceolate
Blade length (cm)	3.8–11.0 × 0.9–3.8	3.5–8.0 × 1.5–3.0	5.0–13.5 × 2.0–3.5	5.7–15.0 × 3.0–5.8
Blade indument on abaxial surface	glabrous, young cinereous-puberulous	glabrous	glabrescent	glabrous
Venation	pinnate to subtripliveined	pinnate to tripliveined	tripliveined	pinnate to subtripliveined
Secondary veins	5 to 7 on each side	5 to 8 on each side	6 to 8 on each side	7 to 9 on each side
Veins on adaxial surface	midrib immersed at the base and flat to the apex, secondary veins flat	midrib slightly immersed at the base and impressed to the apex, secondary veins impressed	midrib immersed at the base and flat to the apex, secondary veins flat	midrib and secondary veins impressed
Veins on abaxial surface	midrib and secondary veins raised	midrib raised, first pair of secondary veins slightly raised, others slightly raised	midrib raised, first pair of secondary veins raised, others slightly raised	midrib raised, first pair of secondary veins raised or slightly raised, others slightly raised
Thickness of midrib (mm)	0.6–1.0	0.5–0.8	0.4–0.8	0.4–0.8
Thickness of first pair of secondary veins (mm)	0.3–0.4	0.1–0.3	0.2–0.4	0.2–0.4
Inflorescence length (cm)	1.4–2.6	1.8–8.0	3.0–13.0	6.0–12.0
Flowers per inflorescence	up to 12	up to 15	up to 60	up to 45
Flower diameter (mm)	2.0–2.2	3.0–4.0	2.7–3.0	3.0–3.5
Flower indument	glabrous	glabrous	glabrous	glabrous to glabrescent
Receptacle shape	subhemispherical, inconspicuous	obconic, conspicuous	obconic, conspicuous	obconic, conspicuous
Receptacle indument	tomentose inside	glabrescent to puberulous inside	puberulous to tomentose inside	puberulous to tomentose inside
Anther whorls I, II and III	4-locular	2-locular	4-locular	4-locular
Filaments of stamens whorl III	3× longer than anther	1–2× longer than anther	1–1.5× longer than anther	1–1.7× longer than anther
Glands of whorl III	glands small 0.4 mm, ovate	glands small 0.4–0.6 mm, globose	glands small 0.5–0.6 mm, ovate	glands large 0.9 mm, elliptical-cubic
Fruit size (cm)	1.1 × 0.8	2.4 × 1.1	1.3 × 0.9	2.0 × 1.2
Cupule shape	obconic, short, covering 1/6 of the fruit	obconic, short, covering 1/5 to 1/6 of the fruit	hemispheric, covering 1/3 to 1/4 of the fruit	turbinate, covering 1/5 to 1/6 of the fruit
Cupule size (cm)	0.9 × 0.6	1.1 × 0.8	1.6 × 1.0	2.3 × 1.2
